# Traumatic In-Situ Fracture of an Etonogestrel Implant (Nexplanon®) Presenting as Altered Palpation Without Bleeding: A Case Report and Review of Literature

**DOI:** 10.7759/cureus.101240

**Published:** 2026-01-10

**Authors:** Quynh Tran, Meryem Soylu, Shagun Tuli, Abraham Madany, Panagiotis Cherouveim

**Affiliations:** 1 Obstetrics and Gynecology, Michigan State University College of Human Medicine, East Lansing, USA; 2 Obstetrics and Gynecology, Hurley Medical Center, Michigan State University, Flint, USA

**Keywords:** contraception, etonogestrel implant, etonogestrel implant fracture, long-acting contraception, nexplanon, nexplanon breakage, trauma

## Abstract

Etonogestrel implant fracture is rare with uncertain clinical impact. We report a traumatic in-situ fracture to highlight evaluation and management. A 27-year-old gravida 3 para 3 (G3P3) presented three months after a fall, noting her upper-arm implant felt “broken.” During examination, palpation demonstrated loss of rod continuity with proximal and distal fragments. She had no abnormal bleeding from the uterus or the implant site. After counseling about uncertain effects on hormone release and efficacy, removal was performed under local anesthesia via two ~5-mm incisions. Two fragments (2.6 cm and 1.4 cm) were retrieved intact, and recovery was uncomplicated. She declined immediate replacement. Traumatic implant fracture may present solely as altered palpation. Clinicians should assess integrity after arm trauma, use imaging if nonpalpable, confirm complete removal, and offer immediate replacement of contraception.

## Introduction

Unintended pregnancies can be prevented by a variety of contraceptive methods. Among those, long-acting reversible contraceptives (LARCs), including intrauterine devices (IUDs) and contraceptive implants, are the most effective ones [1]. LARC failure rates are <1% for both IUDs and subdermal implants, which, in combination with their long-acting nature, increases their popularity [2]. The prevalence of LARC use has increased from 2.4% in 2002 to 8.5% in 2009 to 11.6% in 2012. Of the 11.6% in 2012, 10.3% used IUDs and 1.3% used the implant [3]. 

Nexplanon® (Organon & Co., New Jersey, United States) is an LARC implant commonly used in the United States, which contains 68 mg of etonogestrel (ENG) and measures 4 cm in length and 2 mm in width [4]. The subdermal Nexplanon implant was formerly known as Implanon. Nexplanon’s rod is composed of an ethylene vinyl acetate (EVA) copolymer core at 37% with 15 mg of barium sulfate, which makes the implant radio-opaque and easily localizable on X-ray or ultrasound. Compared to Implanon, the Nexplanon applicator has been simplified to enable insertion with one hand, reducing related complications [5].

Nexplanon works by continuously releasing ENG, a synthetic progestin. Once implanted, serum ENG levels reach a mean level of 265.9 ± 80.9 pg/mL after eight hours and a steady-state release of 200 pg/mL after four to six months. ENG concentrations remain steady for three years and prevent pregnancy by inhibiting ovulation, endometrial proliferation, and thickening cervical mucus [4]. Nexplanon is placed subdermally in the inside of the upper arm by a trained healthcare professional and can be in place for up to three years [3]. Removal requires a small incision after the Nexplanon is located via palpation or ultrasound [4,5].

Known complications include those of improper insertion, incorrect removal, or those related to the implant site. Improper insertion could lead to a protruded implant or insertion into the muscle and/or fascia. The most common complication related to the implant site is pain, which can occur in 2.9% of women. Incorrect removal complications include breakage of the implant, difficulty palpating the implant, the implant being adherent to underlying tissue, and the implant being too deeply inserted. There have also been two cases reported of median nerve injury due to incorrect incision of the arm for removal [6]. A rare complication with only a few reported cases is Nexplanon breakage in situ [7-11].

The true incidence of in-situ ENG implant fracture is unknown. Available evidence is limited, suggesting that this complication is rare but likely underrecognized. Reported risk factors can be broadly categorized as mechanical trauma and repetitive localized stress. Currently, in literature, implant breakage was associated with blunt trauma, weightlifting, picking at the implant area, or was found to be spontaneous or with no recall of trauma. Patients mostly presented with abnormal uterine bleeding, rarely with an irregular shape on palpation, or were found incidentally on routine exam of an asymptomatic patient.

This report describes a rare case of Nexplanon breakage in a 27-year-old female patient in situ due to trauma. We also review the related literature and discuss management considerations.

## Case presentation

A 27-year-old African American female patient (G3P3) presented to the Obstetrics and Gynecology resident clinic with concerns that her Nexplanon was "broken." No other relevant history existed, and she reported no allergies. She reported falling on ice three months prior to the visit and has since felt this. The patient denied any other symptoms associated with Nexplanon breakage, including localized symptoms around the implant (e.g., bleeding, deformity), irregular vaginal bleeding, or changes in menstruation pattern. The implant had been placed two and a half years before the encounter in the nondominant arm using the standard technique. This was her first time using the Nexplanon implant.

**Figure 1:**
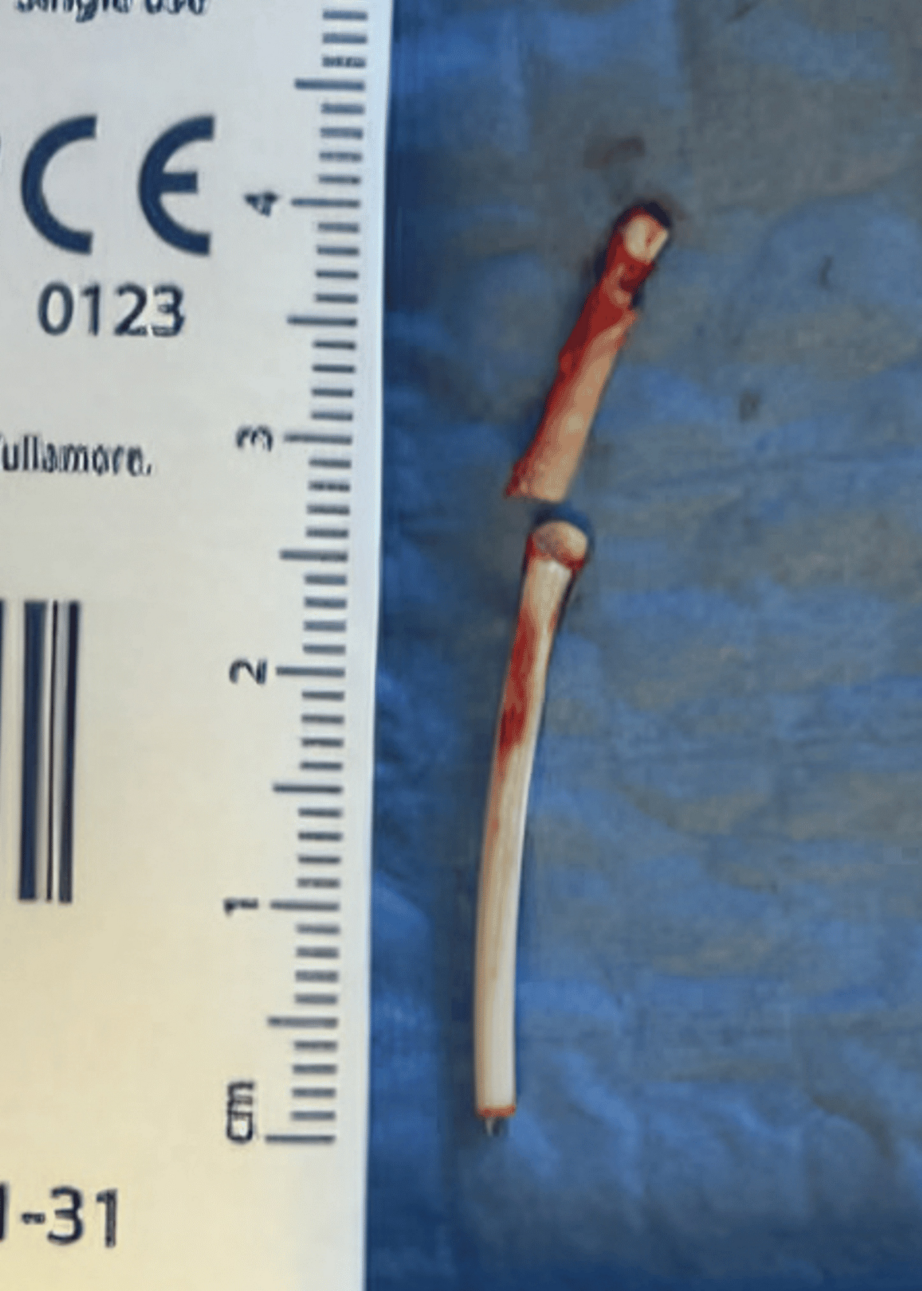
Both parts of the broken Nexplanon implant were removed in their entirety.

On examination, there was a loss of continuity of the Nexplanon implant noted on palpation. The smaller part was proximal, and the longer part was distal. The patient was counseled on the uncertainty of the outcomes and the available evidence on the chances of migration and changes in hormone release, as well as the associated changes in efficacy. The patient opted for the removal of the implant and declined any other form of birth control. Because the Nexplanon could be palpated, no imaging was required prior to removal. It was removed in its entirety with two small (~5 mm) incisions on each end, after utilizing 4 ml of local anesthetic (1% lidocaine). Each piece was removed from its respective incision, and it was confirmed to be removed in its entirety both by palpation and by measuring the two fragments. The Nexplanon rod was noted to be broken in two pieces, 2.6 cm and 1.4 cm in length (Figure 1). The procedure was tolerated well with no complications. 

## Discussion

Fracture of a hormonal implant in situ is rare; the true incidence is unknown, as noted by Hartnell [12]. Howett et al. suggest that a contraceptive implant can be broken at the time of insertion, at the time of removal, or in situ [13]. According to them, breakage at the time of insertion may be due to damage inflicted by the insertion needle to the skin of the implant, which may be weakened and predisposed to fracture when an outside force is applied, even if the core remains whole. Breakage at the time of removal may be due to inadvertent damage caused by the surgical instruments. 

There are previous reports available with cases like our patient’s clinical scenario. In the literature, we found nine reports describing a small number of cases (one to seven cases) and one larger case series conducted by a self-reporting survey, which included 54 patients (Table 1). Pickard and Bacon were the first to describe an in situ Implanon fracture, occurring after blunt trauma, in a patient who presented with prolonged, persistent vaginal bleeding that resolved following implant removal and reinsertion [10]. Agrawal and Robinson reported the first documented case of spontaneous Implanon fracture in the absence of trauma, identified in a patient presenting with heavy menstrual bleeding [11].The first reported Nexplanon fracture, which occurred spontaneously, was a letter to the editor by Elliman around the time Implanon was replaced by Nexplanon [14]. 

**Table 1:** Characteristics of reported LARC implant fracture cases LARC: long-acting reversible contraceptive

Author(s)	Implant Type	Year	Number of Cases	Time Interval	Mechanism (Trauma vs Spontaneous)	Symptoms
Campodonico et al. [7]	Nexplanon	2019	2	8 months 18 months	Blunt trauma and lifting heavy objects	Vaginal bleeding and intermenstrual bleeding
Pickard and Bacon [10]	Implanon	2002	1	5 months	Blunt trauma	Vaginal bleeding
Agrawal and Robinson [11]	Implanon	2003	1	32 months	Spontaneous	Heavy periods
Tomás-Tello and Hodgson [8]	Implanon	2010	2	2 months 34 months	Picked implant x2 (repetitive trauma)	Vaginal bleeding x2
Torres et al. [9]	Implanon	2013	2	24 months 39 months	Spontaneous Grabbed by the arm	Asymptomatic x2
Howett et al. [13]	Implanon	2019	1	13 months	Trauma	Vaginal bleeding
Elliman [14]	Nexplanon	2013	1	20 months	Spontaneous	Vaginal bleeding
Crouthamel et al. [15]	70% (n=14) were Nexplanon, 26% (n=38) were Implanon, and 4% (n=2) were histrelin acetate	2018	54	Time interval between placement and fracture was less than 2 years for 63% (n=34) of cases.	Manipulation (23%, n=12), unintentional trauma (11%, n=6), interpersonal violence (8%, n=4), lifting/carrying (6%, n=3), fracture with removal (6%, n=3), and unknown (47%, n=25)	Bleeding pattern was not altered in 78% (n=42) of cases.
Khatri [16]	Nexplanon	2015	1	36 months	Spontaneous	Asymptomatic
Bentley [17]	6 Nexplanons, 1 Implanon	2013	7	6, 5, 15, 16, 24, 36 months	1 trauma, 6 spontaneous	Vaginal bleeding or symptomatic
Hartnell [12]	Nexplanon	2015	1	7 months	Spontaneous	Asymptomatic

As seen in Table 1, in most of the cases, fractures occurred spontaneously, without a known inciting event, whereas others were secondary to trauma (blunt trauma, repetitive trauma, lifting, picking). The interval time between the insertion and removal varied from five months to 39 months. Symptomatic patients presented with abnormal vaginal bleeding, including heavy menstrual bleeding, spotting, or resumption of menses. In contrast, asymptomatic patients either self-detected an abnormally shaped implant on palpation or had the fracture incidentally identified during a clinical visit, after which the implant was removed.

In the self-reported survey conducted by Crouthamel et al., which included 54 patients, most were 21 years of age or younger (54%), 53% had class 3 obesity, and 70% of the fractured implant cases involved Nexplanon [15]. The time interval between placement and fracture was less than one year for 41% of reported cases, the mechanism of fracture was unknown/spontaneous 47% of the time, while the most common documented reason for an implant fracture was patient manipulation, and the menstrual bleeding pattern was unaffected in most patients [15]. In most of the reviewed cases (Table 1), the implant was broken into almost equal halves. However, Khatri reported a fracture at two sites and unequal pieces [16]. Similarly, Crouthamel et al. reported that 30% of participants had fractures in two separate pieces, 15% reported three or more separate pieces, and 13% were bent but not fractured [15]. 

Disruption in menstrual bleeding pattern was the most common symptom in patients. Pickard and Bacon [10] and Tomás-Tello and Hodgson [8] suggested that the broken implant can result in an inadequate release of the ENG, and thus, be responsible for the irregular bleeding. Torres et al. cited unpublished data from Merck indicating that the release rate of ENG may be increased by rod breakage [9]. However, Rekers, who was the Global Director, Scientific Affairs, Contraception at MSD (Merck Sharp & Dohme), the parent company of Organon at that time, wrote that the in vitro release rate of ENG from the damaged implants increased only slightly compared to the undamaged implants; thus, he concluded that contraceptive efficacy will not be affected by implant breakage [18]. The prescribing information for Nexplanon indicates that disruption of the implant’s structure (e.g., bending or bending) may compromise the controlled ENG diffusion. Increased surface exposure can trigger excess or irregular hormone release, leading to an atrophic endometrium with spotting or breakthrough bleeding. Based on in vitro data, when the implant is broken or bent, the release rate of ENG may be slightly increased [19]. 

Another concern regarding the contraceptive effects of Nexplanon is diminished efficacy if the steady hormone-releasing mechanism is disrupted, although no evidence of this has been found in the literature during the time in which this report was prepared. Bentley described a patient who reported positive home pregnancy tests three days after implant removal, followed by seven days of heavy bleeding with clots; a repeat pregnancy test eight days after removal was negative, suggesting possible contraceptive failure [17]. 

The decision to remove a broken Nexplanon should be made jointly with the patient, and if ongoing contraception is desired, removal with immediate replacement with a choice of contraception offers the most reliable option [20]. Removing damaged implants using the usual pop-out technique is the standard method of removal, but it does carry the risk of needing an additional incision if the implant is completely fractured. It is important to remove the implant in its entirety to ensure the cessation of side effects caused by a retained, broken implant. Careful enquiry and examination, and pre-removal imaging (ultrasound or X-ray) can help locate fragments if they are non-palpable. Current evidence is limited, and further research is needed to clarify the true incidence of breakage, potential risk factors, and their impact on contraceptive efficacy and bleeding profiles.

## Conclusions

Traumatic in-situ fracture of an ENG implant is rare but clinically meaningful. This case illustrates that breakage may follow minor blunt trauma and be asymptomatic apart from an altered palpation profile. Patients should be counseled about the possibility of hormonal implant fracture, advised against excessive manipulation of their implants, and counseled to present for care immediately upon noticing a fracture or altered shape. Because device deformation can unpredictably affect hormone release and complicate removal, clinicians should (i) assess implant integrity when patients report local changes or after arm injuries, (ii) counsel about uncertain effects on efficacy and bleeding with shared decision-making, (iii) use imaging if fragments are not clearly palpable, and (iv) confirm complete removal-considering immediate replacement if ongoing contraception is desired. Broader pharmacovigilance and reporting of breakage events are needed to clarify incidence, risk factors, and optimal management.
